# Localizing on-scalp MEG sensors using an array of magnetic dipole coils

**DOI:** 10.1371/journal.pone.0191111

**Published:** 2018-05-10

**Authors:** Christoph Pfeiffer, Lau M. Andersen, Daniel Lundqvist, Matti Hämäläinen, Justin F. Schneiderman, Robert Oostenveld

**Affiliations:** 1 Department of Microtechnology and Nanoscience, Chalmers University of Technology, Gothenburg, Sweden; 2 NatMEG, Department of Clinical Neuroscience, Karolinska Institute, Stockholm, Sweden; 3 Department of Neuroscience and Biomedical Engineering, Aalto University, Aalto, Espoo, Finland; 4 Athinoula A. Martinos Center for Biomedical Imaging, Department of Radiology, Massachusetts General Hospital/Harvard Medical School, Charlestown, MA, United States of America; 5 Harvard-MIT Division of Health Sciences and Technology, Cambridge, MA, United States of America; 6 Institute of Neuroscience and Physiology, University of Gothenburg and MedTech West, Gothenburg, Sweden; 7 Donders Institute for Brain, Cognition and Behaviour, Radboud University, Nijmegen, Netherlands; Australian Research Council Centre of Excellence in Cognition and its Disorders, AUSTRALIA

## Abstract

Accurate estimation of the neural activity underlying magnetoencephalography (MEG) signals requires co-registration i.e., determination of the position and orientation of the sensors with respect to the head. In modern MEG systems, an array of hundreds of low-*T*_c_ SQUID sensors is used to localize a set of small, magnetic dipole-like (head-position indicator, HPI) coils that are attached to the subject’s head. With accurate prior knowledge of the positions and orientations of the sensors with respect to one another, the HPI coils can be localized with high precision, and thereby the positions of the sensors in relation to the head. With advances in magnetic field sensing technologies, e.g., high-*T*_c_ SQUIDs and optically pumped magnetometers (OPM), that require less extreme operating temperatures than low-*T*_c_ SQUID sensors, on-scalp MEG is on the horizon. To utilize the full potential of on-scalp MEG, flexible sensor arrays are preferable. Conventional co-registration is impractical for such systems as the relative positions and orientations of the sensors to each other are subject-specific and hence not known *a priori*. Herein, we present a method for co-registration of on-scalp MEG sensors. We propose to invert the conventional co-registration approach and localize the sensors relative to an array of HPI coils on the subject’s head. We show that given accurate prior knowledge of the positions of the HPI coils with respect to one another, the sensors can be localized with high precision. We simulated our method with realistic parameters and layouts for sensor and coil arrays. Results indicate co-registration is possible with sub-millimeter accuracy, but the performance strongly depends upon a number of factors. Accurate calibration of the coils and precise determination of the positions and orientations of the coils with respect to one another are crucial. Finally, we propose methods to tackle practical challenges to further improve the method.

## Introduction

Magnetoencephalography (MEG), which measures magnetic fields generated by neural currents, is a tool for non-invasive studies of human brain function. Via sampling of the magnetic fields around the head surface, one can determine neural activity in the brain with high temporal and spatial accuracy. Detection of these weak fields (on the order of 10^−14^ to 10^−15^ T) requires very sensitive magnetometers. State-of-the-art MEG systems employ hundreds of low critical temperature (low-*T*_c_) superconducting quantum interference devices (SQUIDs). [1, 2]

Conventional low-*T*_c_ SQUIDs have to be cooled with liquid helium (4.2 K), which necessitates a well-insulated dewar. This results in a distance between sensor (cryogenic temperature) and head (room temperature) that is on the order of ca. 2–3 centimeters. As magnetic fields decay rapidly with distance, significant gains in signal-to-noise ratio (SNR) would be achieved if the distances between the sensors and the head were reduced.

Fixed-helmet sensor arrays, like the ones used in conventional state-of-the-art MEG systems, allow a high sensor density but are restricted to a single, fixed sensor arrangement. The helmet design and sensor arrangement are chosen to fit the majority of head sizes of a target population, in most cases adults. This excludes some subjects, e.g., with large heads. On the other hand, for subjects with heads significantly smaller than the helmet, a large fraction of the sensors is far away from the head. This is especially problematic for research on young children–a subject group of high interest in neuroscience (e.g. in studies of brain development).

New technologies, such as optically pumped magnetometers (OPMs) and high-*T*_c_ SQUIDs, allow us to get much closer (within a few millimeters) to the head; we refer to these techniques jointly as “on-scalp MEG”. Reducing the distance between sensor and source results in stronger signals and improved spatial resolution, allowing for an improved view on brain activity. Despite their typically higher sensor noise levels, several groups have shown such MEG systems can outperform state-of-the-art systems in terms of extracted information and localization accuracy [3–5] and several measurements have demonstrated their potential [6–11].

On-scalp MEG allows the construction of flexible arrays where the magnetic field sensors are positioned individually or in small modules on the head of the subject. This allows the head size variability in the population to be dealt with by adjusting the sensor array to the individual head–ensuring high SNR independent of head size.

While offering good coverage and improved sensor-to-head distance, adjustable sensor arrays have a practical issue that does not apply to fixed-helmet sensor arrays: how to determine the positions and orientations of all individual magnetometers with respect to the subject’s head. Accurate reconstruction of neural activity from a set of magnetic field measurements from outside the head requires a precise assessment of the location and orientation at which the field is sampled. This is especially important when recording closer to the head [4].

In a flexible system the location and orientation of each individual sensor has to be determined—in contrast to current systems, where the sensor array is fixed and only the whole array needs to be localized relative to the head. We propose to do this using localization coils similar to the ones used in commercial state-of-the-art MEG systems. In commercial systems, three to five localization coils are attached to the head of the subject. These are driven with well-defined, periodic currents at unique frequencies, generating a magnetic dipole field distribution at these frequencies for the different coils. The field strength of each coil detected at each sensor location is extracted from the sensor signals, e.g. by Fourier transform. When the sensor locations inside the helmet and the magnetic moments of the coils are known, the measured field distribution can be used to determine the positions of the coils relative to the sensors and, thereby, the location and orientation of the head with respect to the helmet.

**Fig 1:**
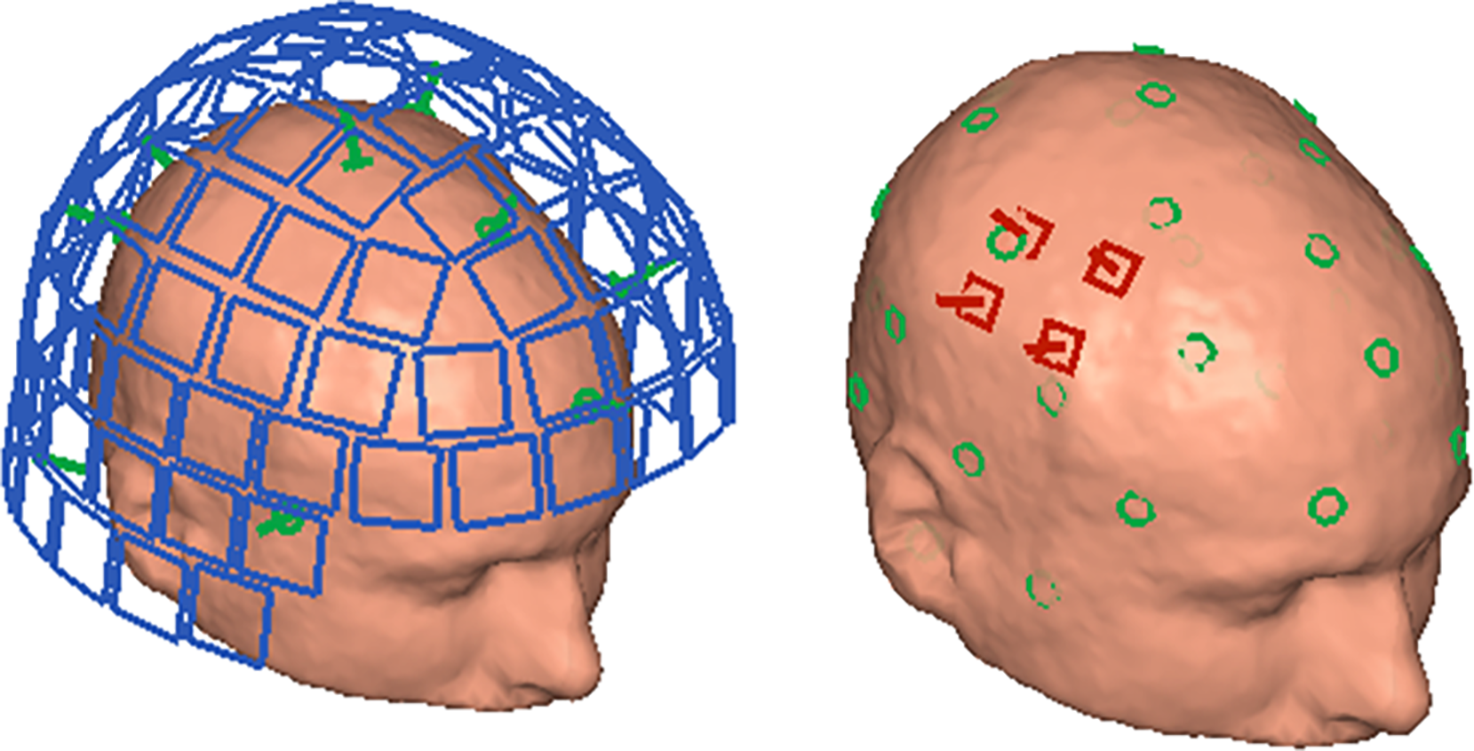
Head localization vs. sensor localization. Left: classical head localization where a large array of sensors (blue; here: the low-*T*_c_ SQUID magnetometer locations of the Elekta Neuromag TRIUX helmet) localizes a set of small, dipolar coils (green) that are attached to the subject’s head. Right: example of the proposed magnetometer localization where an array of dipolar coils (green) is used to localize individual (or small arrays of) on-scalp magnetometers (red).

Instead of localizing the dipolar coils attached to the head with a set of well-known (in respect to position and orientation) sensors, we propose to invert the procedure. We use a well-defined array of dipolar coils to localize the positions and orientations of individual sensors (see Fig 1).

The aim of this study was to simulate the proposed sensor localization procedure with dipolar coils for flexible on-scalp MEG systems and–in order to guide future work—investigate the influence of different parameters on the localization accuracy. The parameters we varied are (i) accuracy of *a priori* knowledge of calibration, position, and orientation of the localization coils, (ii) magnetic moment of the localization coils, (iii) magnetometer noise, (iv) localization time, and (v) number of localization coils.

The sensor localization procedure proposed here is not needed for commercial state-of-the-art MEG systems, which contain a large number of magnetometers and/or gradiometers whose position is fixed and well calibrated. Such systems, however, provide a good reference and can hence be used to test the performance of the proposed procedure in practical experiments. Besides exploring the parameters of the localization coil configuration and the recording details, we investigated how accurately small magnetometer arrays (1 to 10 channels) with a known relative position and orientation can be localized using this approach.

## Materials and methods

All simulations were done on the basis of geometrical data that was acquired from a MEG measurement in a previous study [11]. The involvement of the human volunteer was performed in accordance with the technical development prerogative of the Swedish law for ethical approval of research. The subject was informed about the purpose of the study and consented to participating in the previous as well as in this study.

**Fig 2:**
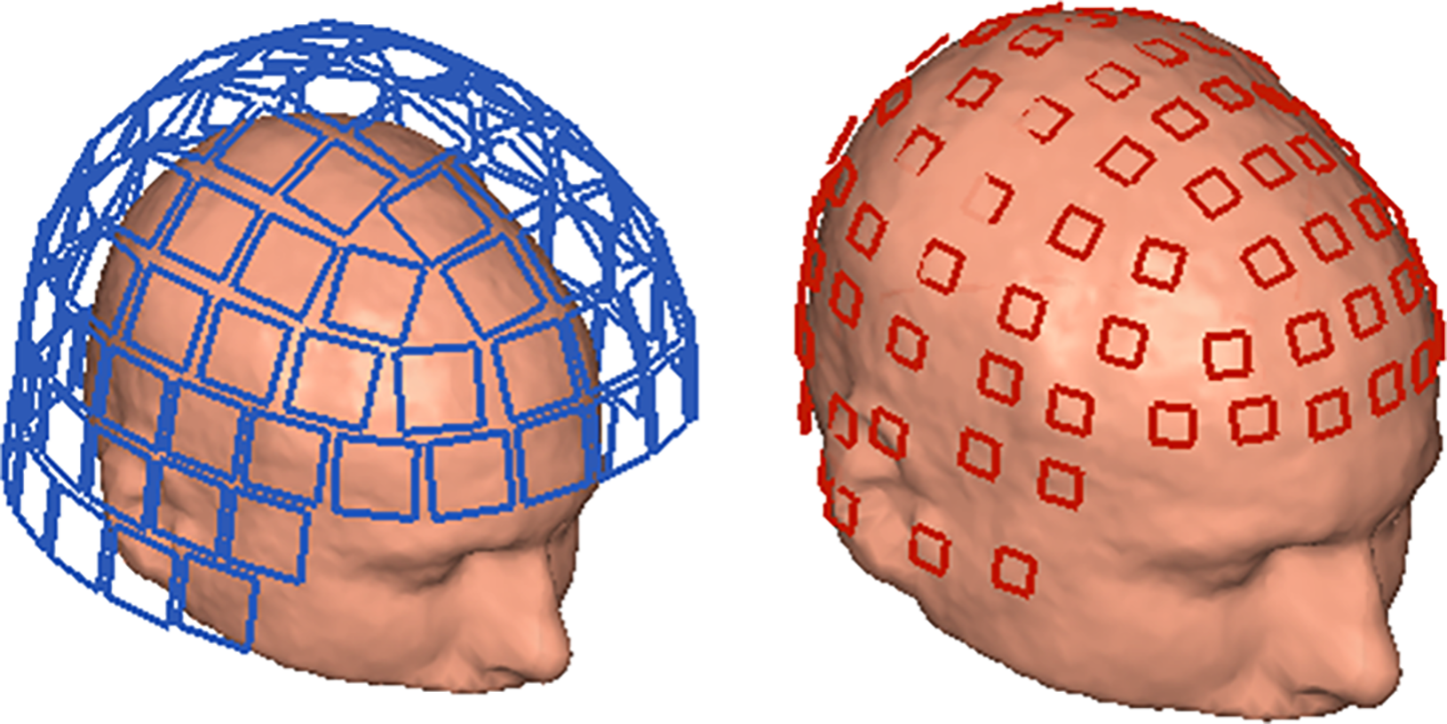
Low-*T*_c_ vs. on-scalp magnetometer positions. Left: Magnetometer positions used for the low-*T*_c_ system (blue; based on the Elekta Neuromag TRIUX). Right: Magnetometer positions used for the on-scalp system (red). Localization is performed for each magnetometer individually.

We performed a number of localizations of individual magnetometers to assess statistical variability. The magnetometer positions were based on the layout of the commercial state-of-the-art low-*T*_c_ SQUID-based Elekta Neuromag “TRIUX” system (hereafter called low-*T*_c_ system). A corresponding set of on-scalp sensor positions were obtained by projecting the TRIUX sensor positions to a distance of 1mm from the subject’s scalp by finding the closest point on the scalp surface and moving it 1 mm in direction of the surface normal at that point. This distance was chosen to account for the minimum separation that can reasonably be achieved with on-scalp sensors [9]. Our particular choice of on-scalp sensor locations was motivated by it being evenly distributed over the head and its similarity to the low-*T*_c_ channel distribution, as shown in Fig 2. Each system has 102 possible magnetometer locations.

**Fig 3:**
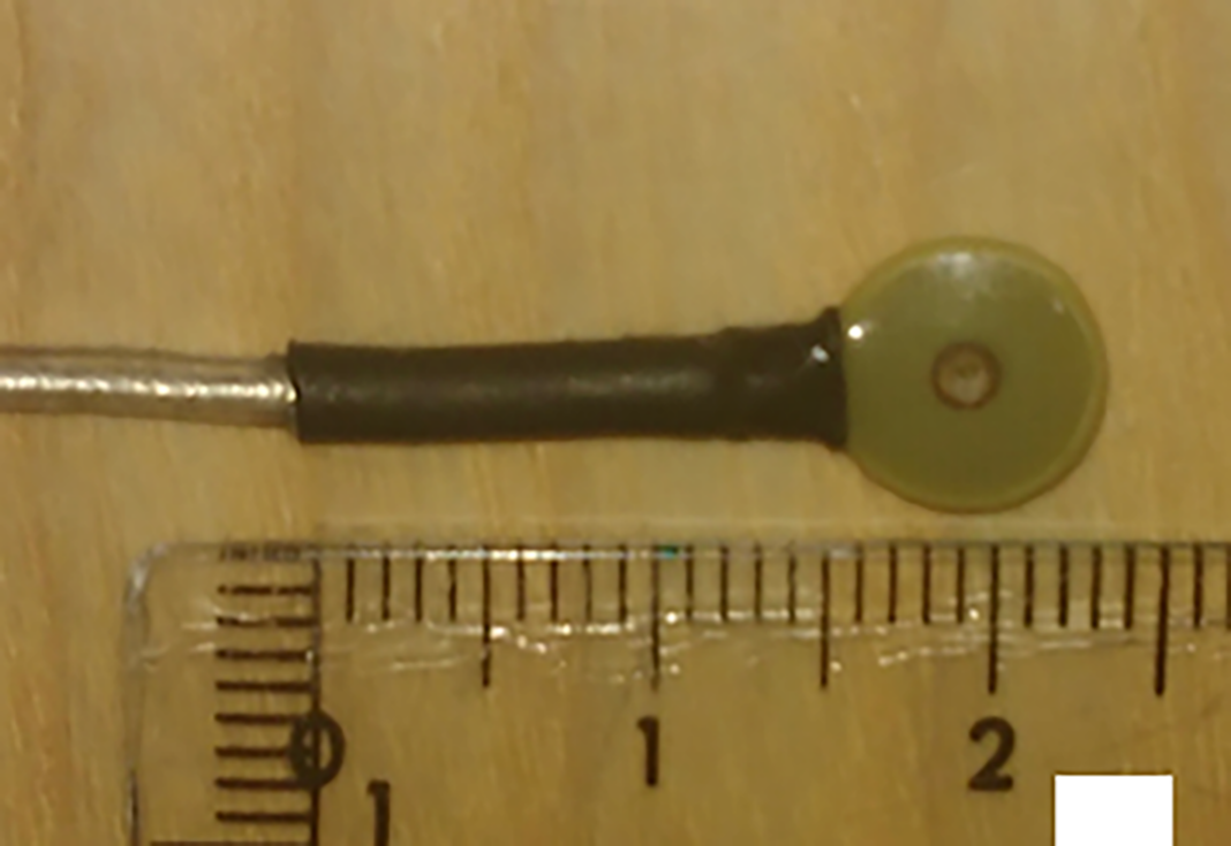
A localization coil used in the Elekta Neuromag TRIUX system (ruler scale is in cm).

We approximated the localization coils as magnetic dipoles to simplify the simulations. This is a valid assumption for coils that are flat and small in size compared to the distance to the magnetometers, like the ones that are typically used in commercial systems (e.g. CTF and Elekta Neuromag TRIUX, see Fig 3).

**Fig 4:**
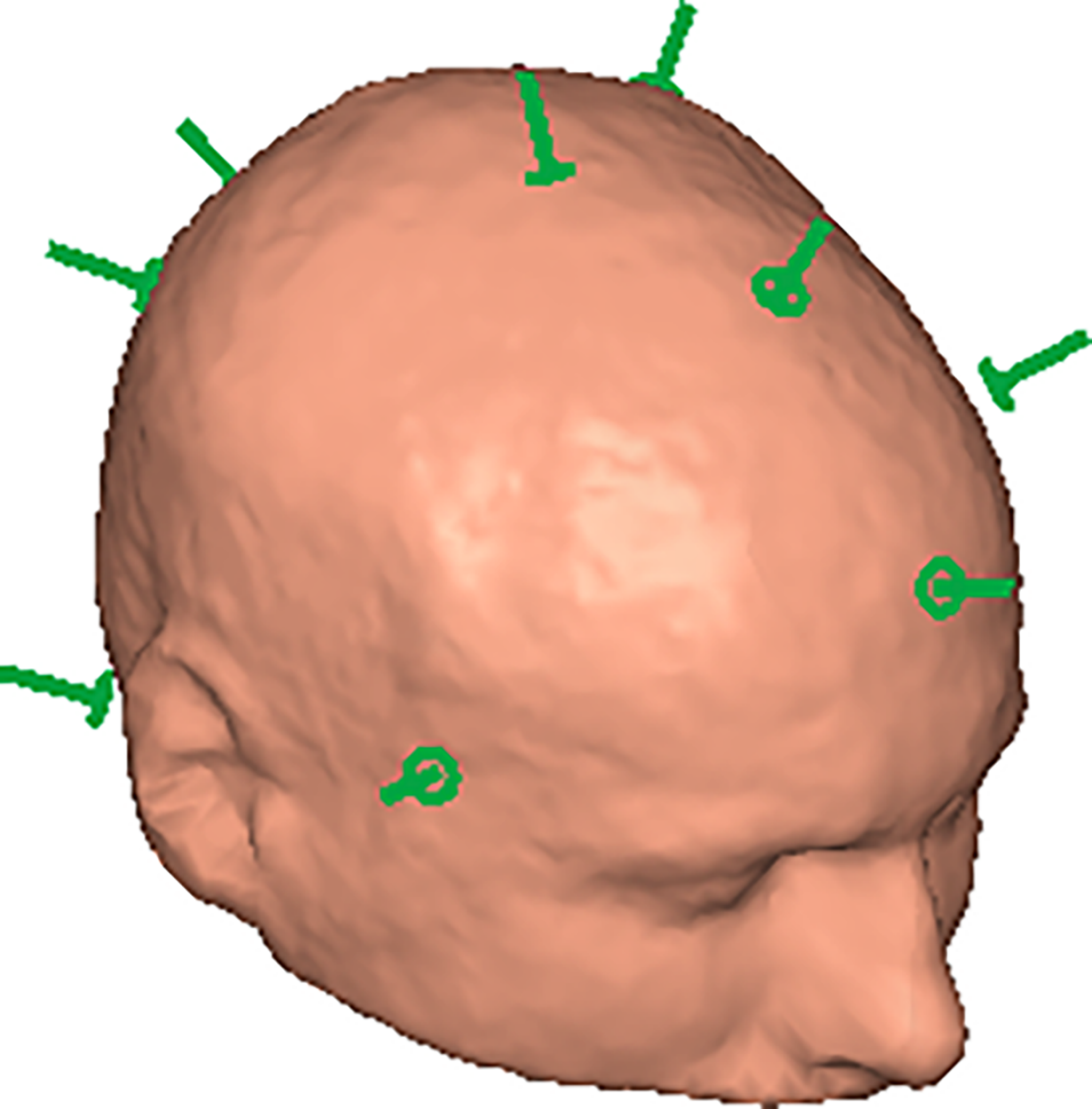
Standard localization coil array (green).

The low-*T*_c_ system allows for 12 localization coils to be configured in software and hardware and comes with localization coils in sets of 10 [11]. Consequently, we have been using 10 localization coils in previous measurements. For most simulations we used 10 locations based on the actual locations from a previous measurement. The coil locations on the head were obtained using a Polhemus Fastrak 3D digitization device (which is part of the standard co-registration procedure when using the low-*T*_c_ system). In optimal conditions, the Fastrak achieves accuracies of <1 mm. For practical measurements, however, position errors of around 2 mm are more common [12]. Fig 4 shows the standard localization coil positions on the subject’s head we used.

### Magnetometer localization

The field from a magnetic dipole that is picked up by a magnetometer, i.e., the forward solution, is given by:

$$S(r_{jk},m_{j},{\hat{n}}_{k})=B(r_{jk},\;m_{j})∙{\hat{n}}_{k}=\frac{\mu_{0}}{4\pi }\left(\frac{3r_{jk}(m_{j}∙r_{jk})}{{\left|r_{jk}\right|}^{5}}-\frac{m_{j}}{{\left|r_{jk}\right|}^{3}}\right)∙{\hat{n}}_{k}$$ (Eq 1)

where r_jk_ is a vector describing the relative position of magnetometer k to the magnetic dipole j, m is the magnetic dipole moment vector and $\hat{n}$ is a unit vector describing the sensitive direction of the magnetometer. For convenience the forward solution is typically defined by the sensor’s spatial sensitivity to (here: magnetic) dipoles, the so-called lead field L_m_.

$$S(r_{jk},m_{j},{\hat{n}}_{k})=B\left(r_{jk},\;\frac{m_{j}}{\left|m_{j}\right|}\right)|m_{j}|∙{\hat{n}}_{k}=L_{m}(r_{jk},{\hat{m}}_{j},{\hat{n}}_{k})∙|m_{j}|$$ (Eq 2)

Localization of a magnetic dipole coil is achieved by fitting a magnetic dipole to the measured/simulated data. The error between the data and the forward solution for the magnetic dipole is the minimized with a non-linear optimization procedure by varying the position, orientation, and moment of the dipole. The error function e used for the optimization is:

$$e(m_{fit})=S_{data}-L_{m}\;m_{fit}$$ (Eq 3)

with S_data_ a vector containing the measured (or simulated) magnetometer signals, L_m_ a magnetometers-by-localization-coils lead field matrix, and m_fit_ a vector containing the dipole moment strengths of the localization coil(s).

For our approach we want to localize individual magnetometers with a set of localization coils instead of localizing coils with a sensor array as in current commercial low-*T*_c_ systems. We therefore exchange the roles of magnetometer and localization coils:

$$m={L_{m}}^{+}*S$$ (Eq 4)

From symmetry follows that ${\left[L_{m}(r,\hat{m},\hat{n})\right]}^{+}=L_{m}(-r,\hat{n},\hat{m})$ i.e., the pseudo inverse of the lead field equals the lead field that would result if the magnetometers were generating the magnetic field and the localization coils sensing it.

By swapping in- and outputs the same dipole fitting functions used in classical head localization can therefore be used to localize a magnetometer with a set of localization coils. A requisite hereto is that positions, orientations, and amplitudes of the localization coils are known. In comparison: classical head localization in commercial full-head systems requires knowledge of position, orientation, and (input) sensitivity of the magnetometers.

The magnetic dipole fits were performed in MATLAB version R2015a (Natick, MA) using the FieldTrip toolbox [13].

### Data generation

The data was generated by applying the forward solution to the signals driving the individual localization coils and summing the resulting magnetic fields that a magnetometer picks up. The localization coils were driven with sine wave signals at different frequencies from 218 Hz up in steps of 7 Hz, in line with the TRIUX system. The driving signal m of coil j is given by:

$$m_{j}(t)=m_{j,0}*sin(2\pi f_{j}t)$$ (Eq 5)

where f is the frequency and m_0_ the amplitude of the magnetic moment.

In addition to the signals from the localization coils, Gaussian noise $N∼N(0,\sigma^{2})$ was added to the magnetometer signals.

$$S(t)=\sum_{j}{(L}_{m}m_{j}(t))+N$$ (Eq 6)

Due to the use of different frequencies, the magnetic moment amplitudes of the individual localization coils can be extracted from the magnetometer signal through Fourier transform or phase-locked detection [14].

To reduce computation time, we chose the real positions of the localization coils as starting points for the optimization. This represents a fitting with an initial guess (i.e., from the digitization point) similar to the way the digitized locations of the coils are used in conventional head localization. Alternatively, a grid-search could be performed to find an initial starting point for the non-linear fit.

We localized the magnetometers individually and defined the localization error as the difference between the actual magnetometer location and the location obtained from the fit. The localization errors were then averaged over all magnetometers; this entire process was executed for each of the two systems.

#### Signal-to-noise ratio

To investigate the influence of the signal-to-noise ratio (SNR) on the localization accuracy, we performed simulations with different magnetometer noise levels and magnetic moment amplitudes. The noise was varied from σ = 0.1 to 500 fT/Hz^1/2^, the magnetic moment amplitudes from m_j,0_ = 0.25 nAm^2^ to 250 nAm^2^.

Since the Fourier transform is used for localization, the length of the recorded data also determines the SNR. Random noise reduces by averaging with the square root of the data length. If the signal does not change, the SNR therefore increases with increasing data length. Head localization with commercial systems is limited by practical considerations: averaging over time acts as a low pass filter, thereby limiting the speed with which movements of the head relative to the sensor array can be detected. Head localization is therefore typically performed with a sliding window of about one second.

Recently experimental procedures for low-*T*_c_ MEG were proposed where the relative positions of the head of the subject with respect to the sensors are restricted by the use of a 3-D printed or molded head cast [15, 16]. Since head movements are minimized by such an approach, the data length used for localization can be increased significantly to achieve higher SNR. Although on-scalp MEG systems are still at an experimental stage, we anticipate that these systems will have the sensors fixed relative to the head, as with EEG electrode caps or with the 3-D printed MEG head cast procedure (as in [16]). Therefore we expect that the head and sensors will not move relative to each other and that long stretches of data can be acquired with a constant localization coil signal.

To compare our simulations to present-day commercial MEG systems, we used data segments of one second with a sampling frequency of 1 kHz. Additionally, we performed a simulation with 10-minute data length to test how much the localization accuracy could be improved in systems where head movements are not a concern.

In the following simulations we used typical noise values for low-*T*_c_ and on-scalp magnetometers. While low-*T*_c_ SQUID magnetometers with noise levels around 0.15 fT/Hz^1/2^ have been reported [17], these are, to date, not standard in MEG. Magnetometers in commercial systems like the TRIUX have typical noise levels of around 3 fT/Hz^1/2^. While there have been several reports of OPMs and high-*T*_c_ SQUIDs with noise levels as low as a few fT/Hz^1/2^ [18, 19], such high-performance sensors have only been produced in small quantities. We therefore employed a more conservative noise level of 20 fT/Hz^1/2^ that may be more typical of commercially-produced on-scalp sensors. We furthermore used 10 nAm^2^ for the magnetic dipole moment amplitude, consistent with the TRIUX system.

#### Calibration, position and orientation

As mentioned above, localizing the magnetometers requires knowledge of the magnetic moment, position and orientation of the localization coils. To investigate the influence of these parameters on the localization accuracy we simulated different types of inaccuracies in these parameters.

Instead of varying the magnetic moment we applied an error to the calibration values of the localization coils. In practice, the operator of a MEG system has control over the current applied to the localization coils, but has to rely on the accuracy of the calibration value for the conversion from current to magnetic moment. Errors in the calibration value thus translate to the magnetic moment. We therefore performed simulations with random calibration errors in a range of e_calib_ = ±0.1 to ±10%.

We furthermore simulated errors in the position and orientation of the localization coils. First, we added random position errors of e_pos_ = ±0.5 to ±5 mm in three orthogonal directions. Subsequently, we performed simulations with randomly applied orientation errors by rotating the localization coils e_ori_ = ±1 to ±10 degrees around the X-, Y-, and Z-axes.

We simulated each of the aforementioned error cases (calibration, position and orientation) ten times. The errors were then averaged over those repetitions as well as over the magnetometer locations.

#### Localization coil arrays

We also simulated the effects of using different numbers of localization coils. Here we followed two approaches corresponding to different experimental procedures for MEG recordings.

**Fig 5:**
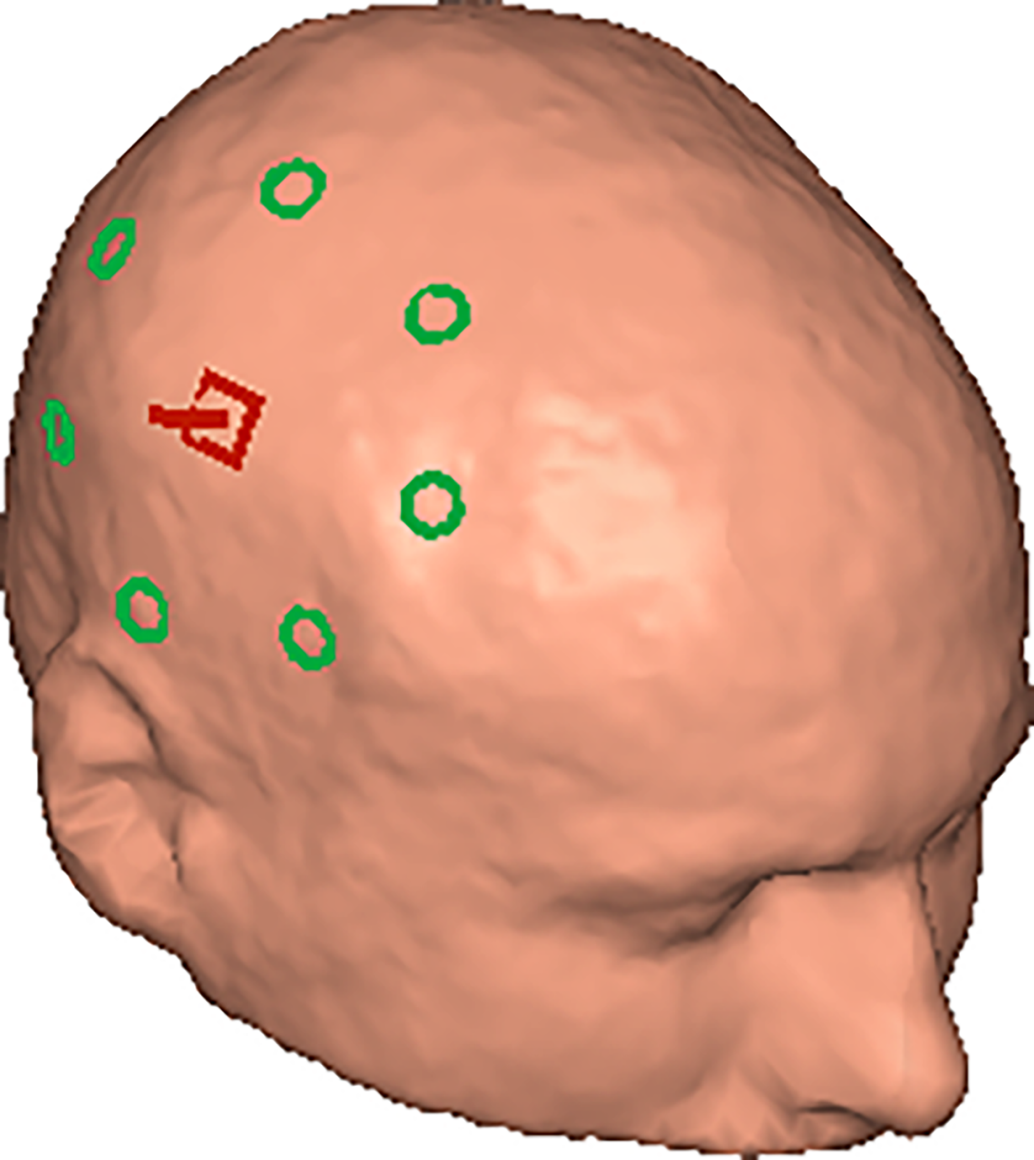
Local localization coil arrays. Placement of localization coils (green) in a small (here: 7-coil) array around an on-scalp magnetometer position (red).

First, we assumed the case of recording the neuromagnetic field only on a small area of the head, e.g., to measure the dipolar pattern of a single source. In such a case, the localization coils can be placed close to that area to ensure good signals from all coils. We realized this by constructing a ring of localization coils with a 5 cm radius around each of the magnetometer positions (see Fig 5) and localizing all the magnetometers falling inside that ring. We simulated such ring-shaped localization arrays at all 94 sensor locations where such a ring fit on the head. This test was furthermore carried out with 4 to 10 localization coils.

**Fig 6:**
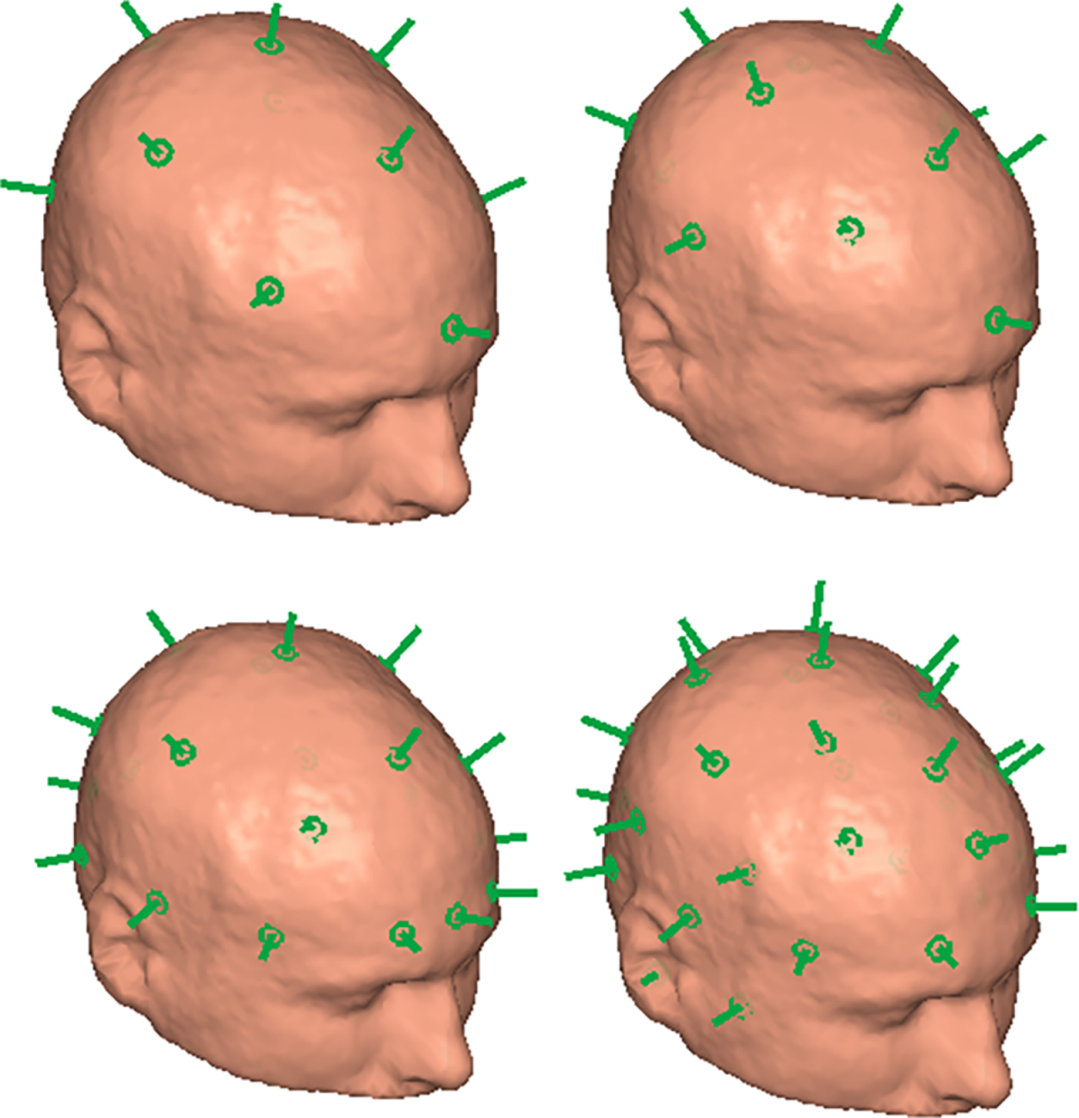
Full-head localization coil arrays. Localization coil placement in 10- (top-left), 12- (top-right), 21- (bottom-left) and 32- (bottom-right) coil full-head arrays.

Second, we assumed that MEG signals on the whole head are to be recorded in a single session. In such a measurement, a single localization array is required that should provide good coverage over the whole head. We simulated whole head arrays of 10, 12, 21 and 32 localization coils (see Fig 6). The layouts are based on the 10–10 EEG system locations and chosen with the aim of uniform coverage. With such whole-head localization arrays, we localized all magnetometer positions and averaged the localization error.

#### Small magnetometer arrays

**Fig 7:**
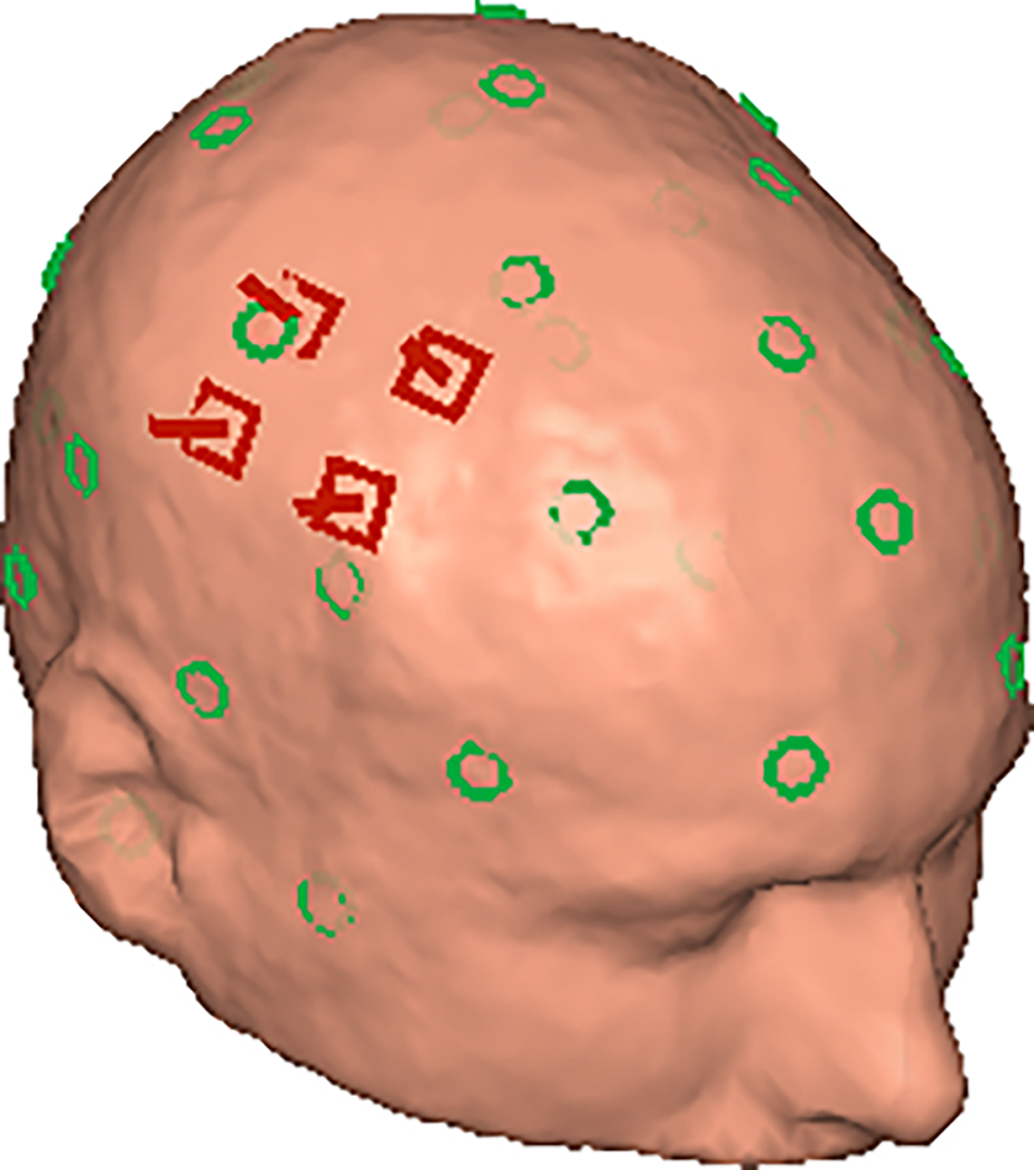
Small magnetometer array. Example of a small array of on-scalp magnetometers (red) that are fit as a group with a 32-coil localization array (green).

Finally, we investigated how a small array of magnetometers would compare in terms of localization accuracy. OPMs can be designed such that a single vapor cell provides 4 measurement points on the corners [20] and high-*T*_c_ systems can be composed of several small cryostats that each contain multiple, tightly packed sensors. If the relative positions and orientations of a set of magnetometers are known, they can be fitted as a group, potentially improving accuracy. We simulated this for arrays of 1 to 10 magnetometers, where we used the nearest neighbors of each magnetometer to form a mini-array (see Fig 7). We determined the average localization error as a function of the numbers of magnetometers in the mini-array.

## Results

### Signal-to-noise ratio

**Fig 8:**
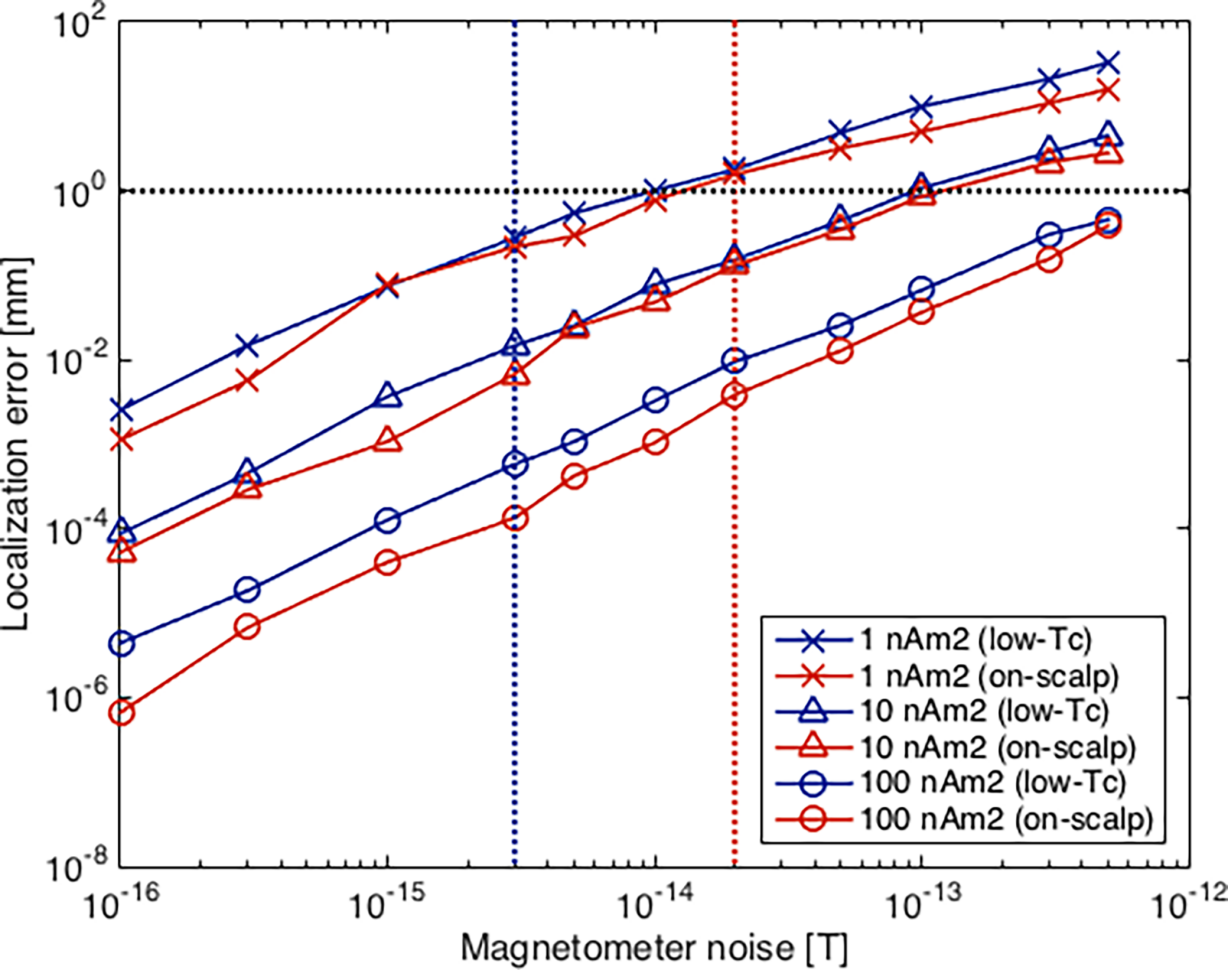
Localization error vs. SNR. Mean localization error with low-*T*_c_ (solid blue) and on-scalp (solid red) systems for different magnetometer noise levels and localization coil magnetic moments (both axes plotted logarithmic). Typical noise levels are marked by vertical blue (low-*T*_c_) and red (on-scalp) dotted lines. The horizontal black dotted line indicates 1 mm localization error.

The results of our simulations with varying noise levels and magnetic moment for the low-*T*_c_ and on-scalp systems are presented in Fig 8. Both magnetic moment and magnetometer noise have a significant impact on the localization error. The accuracy decreases rapidly with higher noise levels, while the magnetic moment shows the opposite effect. This shows that the SNR is the defining characteristic for this localization approach. At identical noise levels and magnetic moments, the on-scalp system shows better accuracy.

Considering the typical sensor noise levels, a typical low-*T*_c_ system with 3 fT/Hz^1/2^ noise level and 10 nAm^2^ magnetic moment would achieve a magnetometer localization accuracy of around 0.01 mm, while a typical on-scalp system with the same magnetic moment and 20 fT/Hz^1/2^ noise level would reach approximately 0.1 mm.

**Fig 9:**
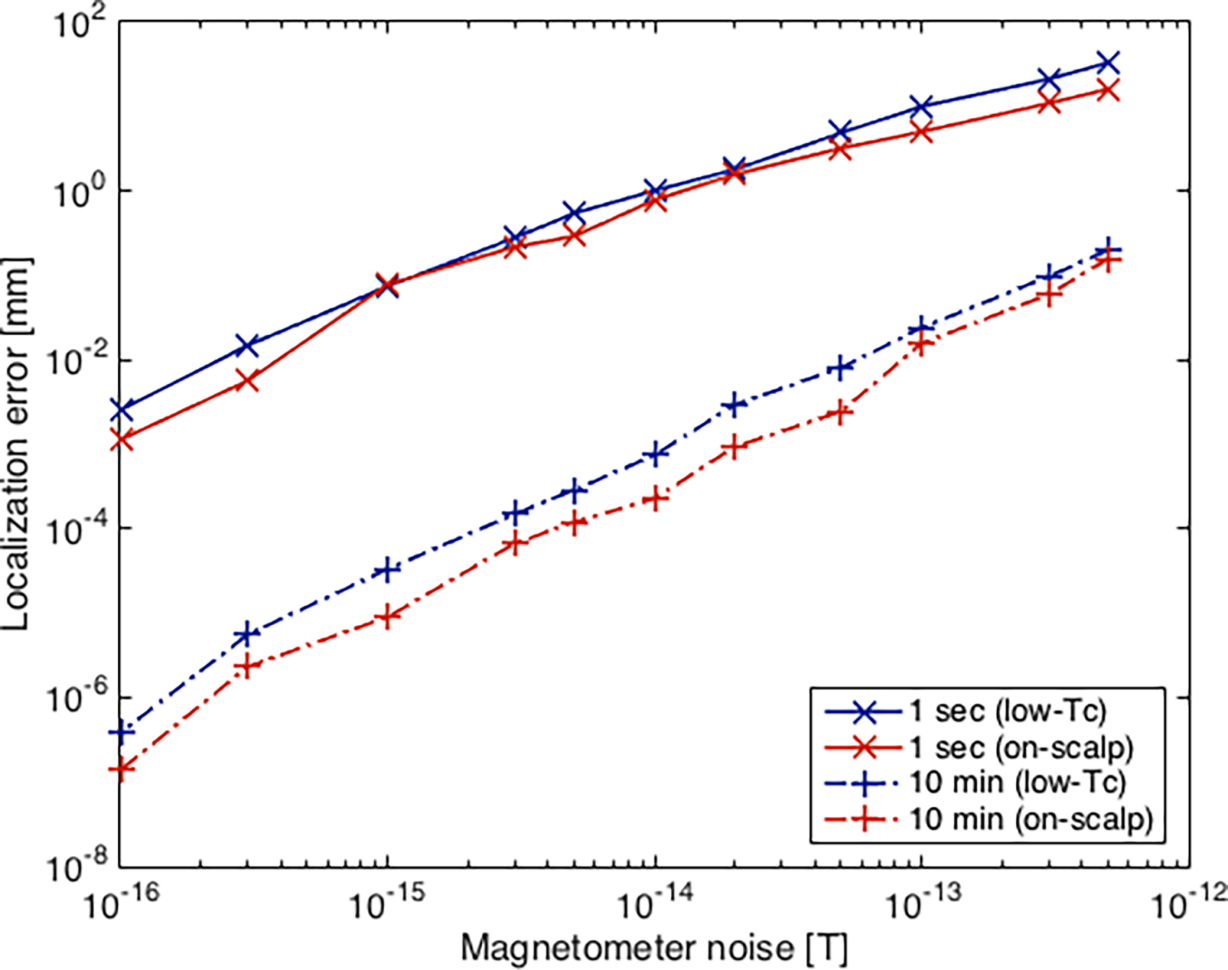
Localization error vs. SNR for different measurement time. Mean localization error with low-*T*_c_ (solid) and on-scalp (dashed) systems for different data lengths. Magnetic moment = 1 nAm^2^. Both axes are plotted logarithmic.

The results for longer measurement time are presented in Fig 9. Computing the localization coil topographies over 10 minutes of data reduces the localization error compared to one second of data. As expected, this demonstrates that longer localization measurements are preferable in terms of accuracy.

### Calibration, position and orientation

**Fig 10:**
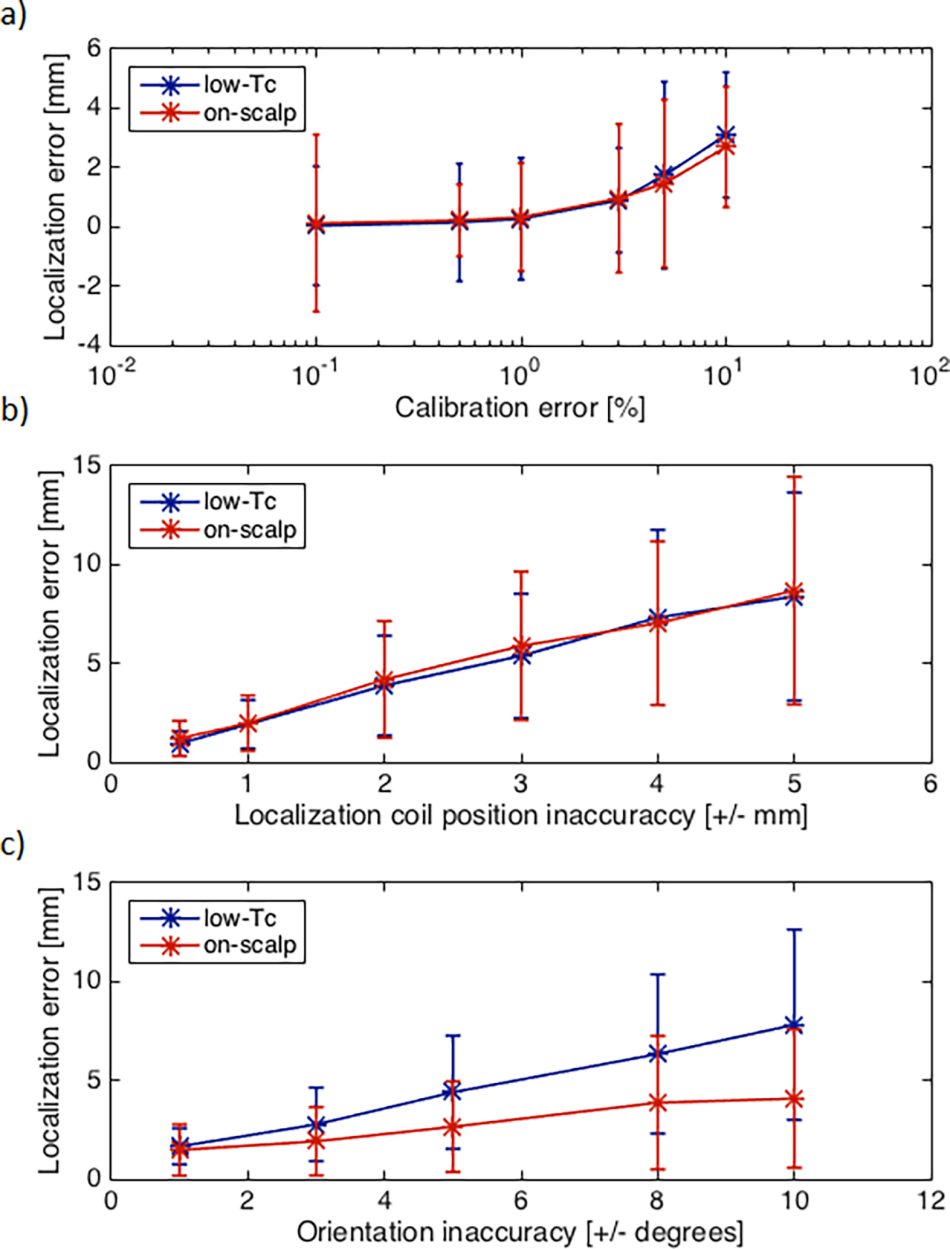
Localization error vs. *a priori* errors. Mean localization error with low-*T*_c_ (blue) and on-scalp (red) systems for different a) calibration error ranges, b) position inaccuracies ranges, and c) orientation error ranges. Magnetic moment = 10 nAm^2^, on-scalp magnetometer noise = 20 fT/Hz^1/2^, and low-*T*_c_ magnetometer noise = 3 fT/Hz^1/2^. Error bars indicate one standard deviation.

Fig 10 shows the magnetometer localization errors that result from imprecise assumptions on the localization coil calibration, position, and orientation.

For typical calibration errors of less than ±1%, the simulations show that both systems localize with an accuracy better than 1 mm. Higher calibration errors can lead to localization errors of >3 mm.

The magnetometer localization error shows a strong, approximately linear dependence on the localization coil position inaccuracy. With position errors on the order of 2 mm (i.e., the typical precision of the digitization device used [12]), mean localization errors of around 4 mm can be expected.

The magnetometer localization accuracy as a function of the localization coil orientation inaccuracies shows, in contrast to calibration and position inaccuracies, a clearly different behavior in the low-*T*_c_ and on-scalp systems. Especially for higher orientation inaccuracies the low-*T*_c_ system shows a stronger increase in the localization error compared to the on-scalp system. For small orientation errors the localization accuracies converge. The difference results from the larger distance between magnetometers and localization coils in the low-*T*_c_ system as compared to the on-scalp system.

### Localization coil arrays

**Fig 11:**
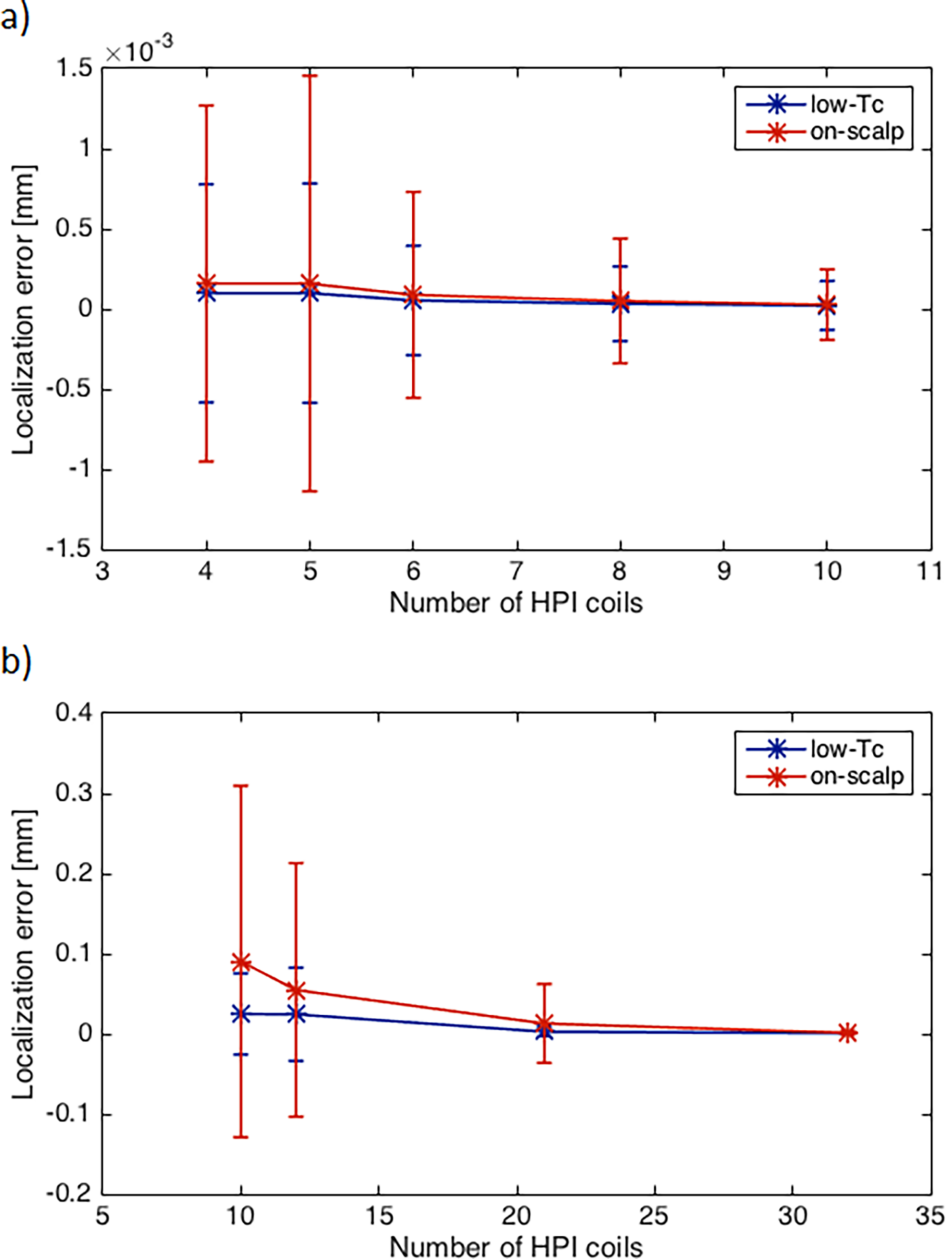
Localization error vs. number of coils. Mean localization error with low-*T*_c_ (blue) and on-scalp (red) systems for different numbers of localization coils a) in small, local localization arrays b) in full-head localization arrays. Magnetic moment = 10 nAm^2^, on-scalp magnetometer noise = 20 fT/Hz^1/2^ and low-*T*_c_ magnetometer noise = 3 fT/Hz^1/2^. Error bars indicate one standard deviation.

Fig 11, panel a) shows how the localization errors change with an increasing number of localization coils in a small, ring-shaped localization coil array. The on-scalp system shows slightly higher localization errors and both systems show decreasing localization errors when using more than five coils. With localization errors of around 10^−4^ mm (even with four localization coils) both systems show more than sufficiently high accuracy.

The localization error as a function of the number of localization coils arranged as full-head arrays can be seen in Fig 11, panel b). Both systems show small localizations errors (<0.1 mm) even with just 10 localization coils. This improves further with increasing number of coils. The on-scalp system benefits especially from increasing the number of localization coils.

### Magnetometer arrays

**Fig 12:**
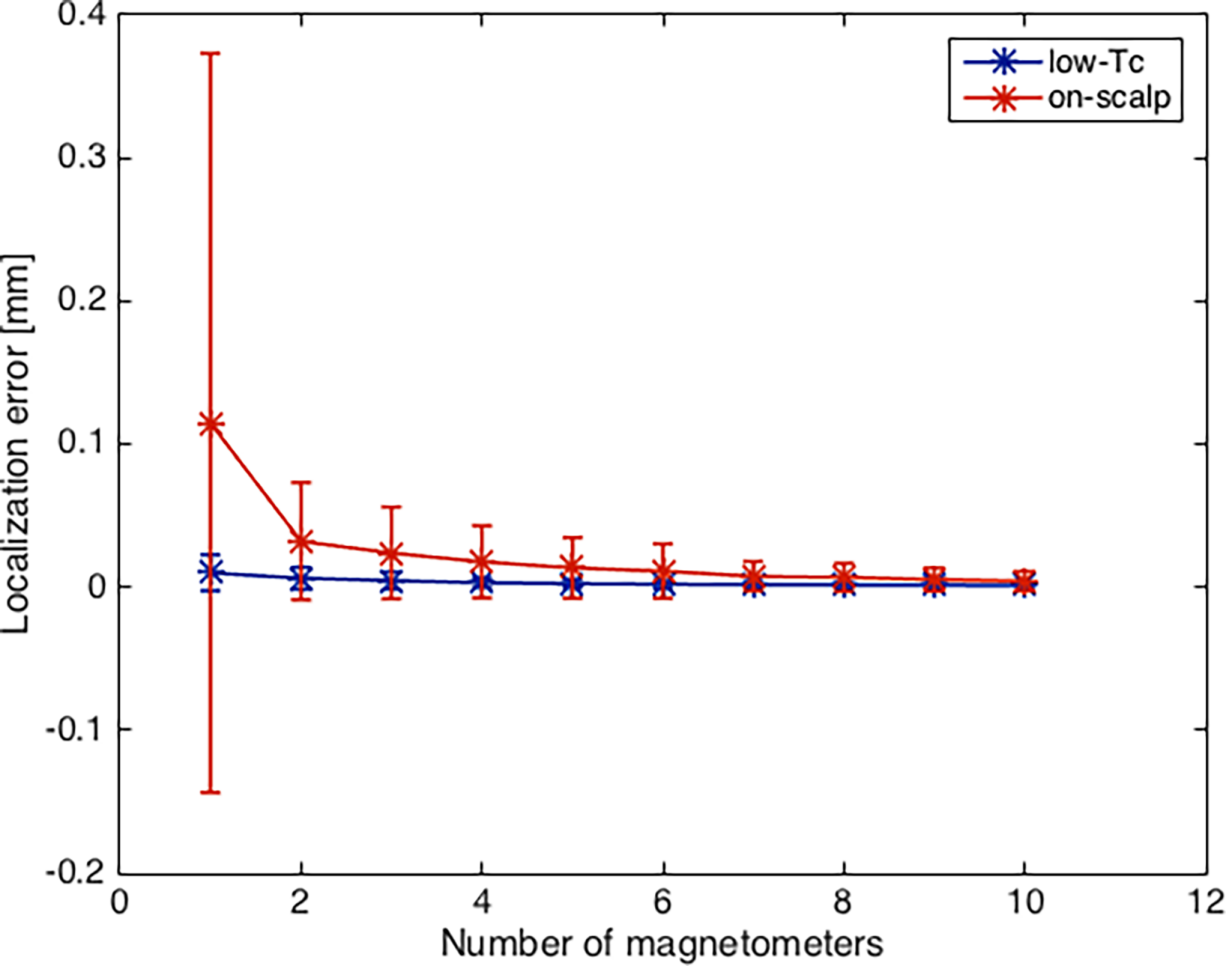
Localization error vs. number of magnetometers. Mean localization error with low-*T*_c_ (blue) and on-scalp (red) systems for different numbers of magnetometers fitted as a group. Magnetic moment = 10 nAm^2^, on-scalp magnetometer noise = 20 fT/Hz^1/2^ and low-*T*_c_ magnetometer noise = 3 fT/Hz^1/2^. Error bars indicate one standard deviation.

As expected, fitting multiple magnetometers with known relative positions and orientations as a group results in higher accuracy (see Fig 12). The reduction in localization error is strongest for the first few additional magnetometers and slowly flattens out. The on-scalp system benefits more from the use of small magnetometer arrays than the low-*T*_c_ system. When fitting 10 magnetometers combined they show a reduction of the localization error by a factor of approximately 27, and 9 respectively, as compared to localizing individual sensors.

## Discussion

Our results demonstrate the feasibility of the proposed approach to localize individual magnetometers with an array of localization coils. Our simulations show that for on-scalp systems we expect better accuracy than for low-*T*_c_ systems due to the closer proximity between the sensors and the localization coils. At identical noise levels they show smaller magnetometer localization errors, less sensitivity to inaccuracies in the assumed localization coil orientations, and a stronger improvement with increasing number of localization coils in small, local ring-arrays. Practically though, currently available on-scalp magnetometers have higher noise levels than low-*T*_c_ SQUIDs. With typical noise levels (low-*T*_c_: 3 fT/Hz^1/2^; on-scalp: 20 fT/Hz^1/2^), the localization of magnetometers in low-*T*_c_ systems is better than the localization of on-scalp magnetometers in most cases.

In the following we discuss the individual results from above and a limitation of the simulations due to the use of dipole approximations.

### Signal-to-noise ratio

The Signal-to-Noise ratio has shown to be a key factor. Since it is hard to significantly decrease the sensor noise for on-scalp sensors, a sensible step is to increase the strength of the localization signal. However, the localization coil field strength is limited by the dynamic range of the sensors. To optimize the SNR, the localization signal should therefore be chosen such that the magnetic field is maximal at the magnetometers without saturating them. Imagining an ideal system, one could consider implementing active control of the localization signal (with sensor signal as an input) to maximize the SNR while avoiding sensor saturation.

Another way to improve the SNR is to increase the recording and averaging time. Here it is important to remember the trade-off between recording time for localization and the ability to register head movements: longer averaging improves SNR but limit the speed with which movements between the head and sensors can be detected. This might especially be an issue when trying to localize epileptic foci in patients that experience strong seizures [21]. However, there are ways to avoid the head movement problem. With custom-designed head casts, for example, the head of a subject can be fixed inside a conventional MEG dewar [14]. While this improves the SNR, the physical constraint of the subject’s head might cause other problems such as claustrophobia, discomfort and risk of injury (e.g. in case of seizure). An alternative approach is to fix the system to the head, instead of the head to the system. This requires the system to be light enough for the head of a subject to hold it, as for example with an EEG cap. For low-*T*_c_ systems that is (with current technology) impossible, but for on-scalp sensors it is conceivable that this can be implemented. While the sensor locations can be determined relatively simple in such cap systems (e.g. with measuring tape as in EEG systems) a more sophisticated co-registration technique would still be necessary to provide the sensor orientations which are–in contrast to EEG–crucial for reconstructing neural activity in MEG.

### Calibration, position and orientation

The strong dependence of the localization accuracy on the calibration of the localization coils shows the importance of accurate calibration of the localization coils. As mentioned before (see Methods), head localization requires exact knowledge of the position, orientation, and calibration factor of the magnetometers, but not the localization coils. As commercial systems employ hundreds of magnetometers to localize a few coils, it is not necessary to know the magnetic dipole strength of the localization coils as these are fitted along with the position and orientation. For this reason, the localization coils used in commercial systems are typically not calibrated and can easily be swapped out for new ones in case they break due to daily handling. For on-scalp magnetometer localization our results suggest that they should be carefully and consistently calibrated.

Like the calibration, the position of the localization coils has a strong influence on the accuracy of the localization. As such, the more accurately the localization coils positions relative to the head can be determined, the better. The standard technology used for localizing localization coils on the head–the Polhemus Fastrak–offers relatively good accuracy. In the last years optical localization and 3-D scanning technologies have emerged that might provide better accuracy and faster recording procedures, while avoiding the potential issue of localization coil movements due to physical contact with a digitizer stylus [22–24].

**Fig 13:**
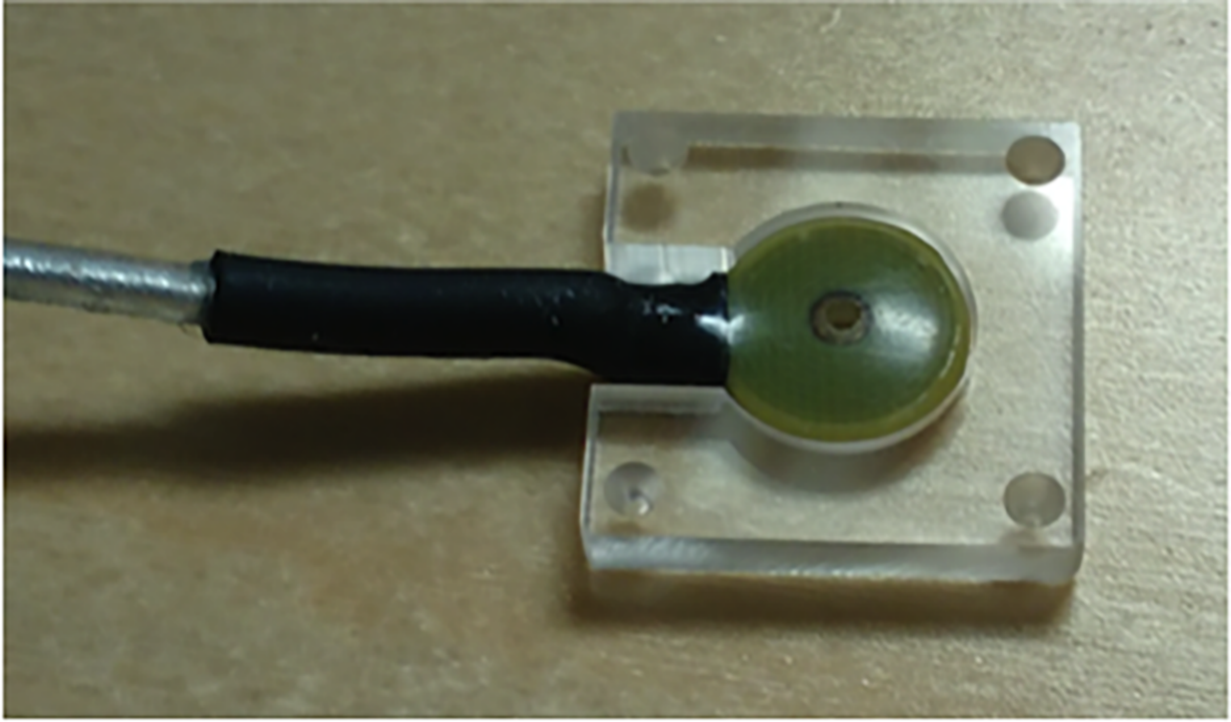
An orientation-digitizable dipolar coil prototype. Photograph of a localization coil mounted on a plate for accurate determination of the coil orientation. At the corners are small indentions for the tip of the Polhemus stylus to assist in the digitization.

An issue to be addressed is the accuracy of determining the localization coil orientation. The small localization coils like the ones used in commercial systems (e.g., Elekta Neuromag TRIUX, which is approximately the size of an EEG cup electrode) offer no simple way to determine their orientation. To improve on this problem, the design of the localization coils could therefore be revised. By fixing a coil to a larger plate, for example, it would be possible to determine the coil orientation by digitizing the plate with several points (given that the orientation of the coil relative to the plate is known). A prototype for such a coil setup can be seen in Fig 13. The setup could be further improved by, e.g., using fabrication techniques such as multilayer printed circuit boards to develop smaller, more reproducible coils where the plate for determining the orientation is integrated.

Another way to determine the orientation could be to use multiple coils with fixed relative orientations and positions. Knuutila et al., for example, mounted three coils together onto a fiberglass plate [25] and Adjamian et al., used five coils attached to a bite-bar for improved co-registration [26]. By reducing the number of independent variables a fixed localization coil arrangement can be used to localize sensors even without accurate knowledge of the coil orientations.

To ensure accurate and maximally stable coil positions and orientations the coil array could further be supported by a mechanical support structure. A simple, thin head-mold made out of, e.g., plaster could be made for a specific subject. Coils could then be glued to it to improve stability.

### Localization coil arrays

Both local and full-head localization coil arrays demonstrated good localization accuracy for the magnetometers, even with few localization coils. The design of the arrays that we simulated was based on simple, intuitive ideas; these could be further optimized to achieve better results.

Increasing the number of localization coils clearly improves the magnetometer localization accuracy. For on-scalp systems a large number of coils could, however, bring new problems, especially in case of full-head arrays. The localization coils used in commercial systems are typically around 1–2 mm thick. That means the magnetometers have to be placed either at locations where there is no coil, or have to be placed further away from the head. Since the reduced sensor-to-scalp distance is one of the main benefits of on-scalp MEG, as compared to conventional, MEG, it is preferable to avoid moving the sensors away from the head. More coils would thus result in fewer possibilities for magnetometer placement. When designing a full-head localization coil array, this should be kept in mind.

### Magnetometer arrays

We observed that fitting small arrays of magnetometers results in improved localization accuracy compared to individually localizing each magnetometer. In the extreme case this can be compared to commercial low-*T*_c_ MEG systems, where a single, fixed 100+–channel sensor array can be fitted using a few localization coils. Chella et al., for example, used a similar method to calibrate a 153-channel low-*T*_c_ MEG system with a 31-coil phantom [27]. For flexible on-scalp systems this is not possible as the position of each channel will be different in each measurement/subject. For several small arrays with a few sensors each, on the other hand, this might be an option for improving the localization of the arrays relative to the head.

Fitting magnetometer arrays in unison should only be done if there is high confidence in the accurate relative positions and orientations of the sensors to each other. If the geometry of the array were inaccurate, the errors would severely affect the localization accuracy, similar to position/orientation errors of the localization coils. This could possibly lead to joint localization that is worse than the individual sensor case.

### Dipole approximation

We used magnetic dipoles to approximate the localization coils for faster computation. Since the coils were approximated both in data generation and in localization they match exactly. In practical measurements, however, this would not hold true. It is therefore necessary to mind the limitations of approximating coils with magnetic dipoles.

**Fig 14:**
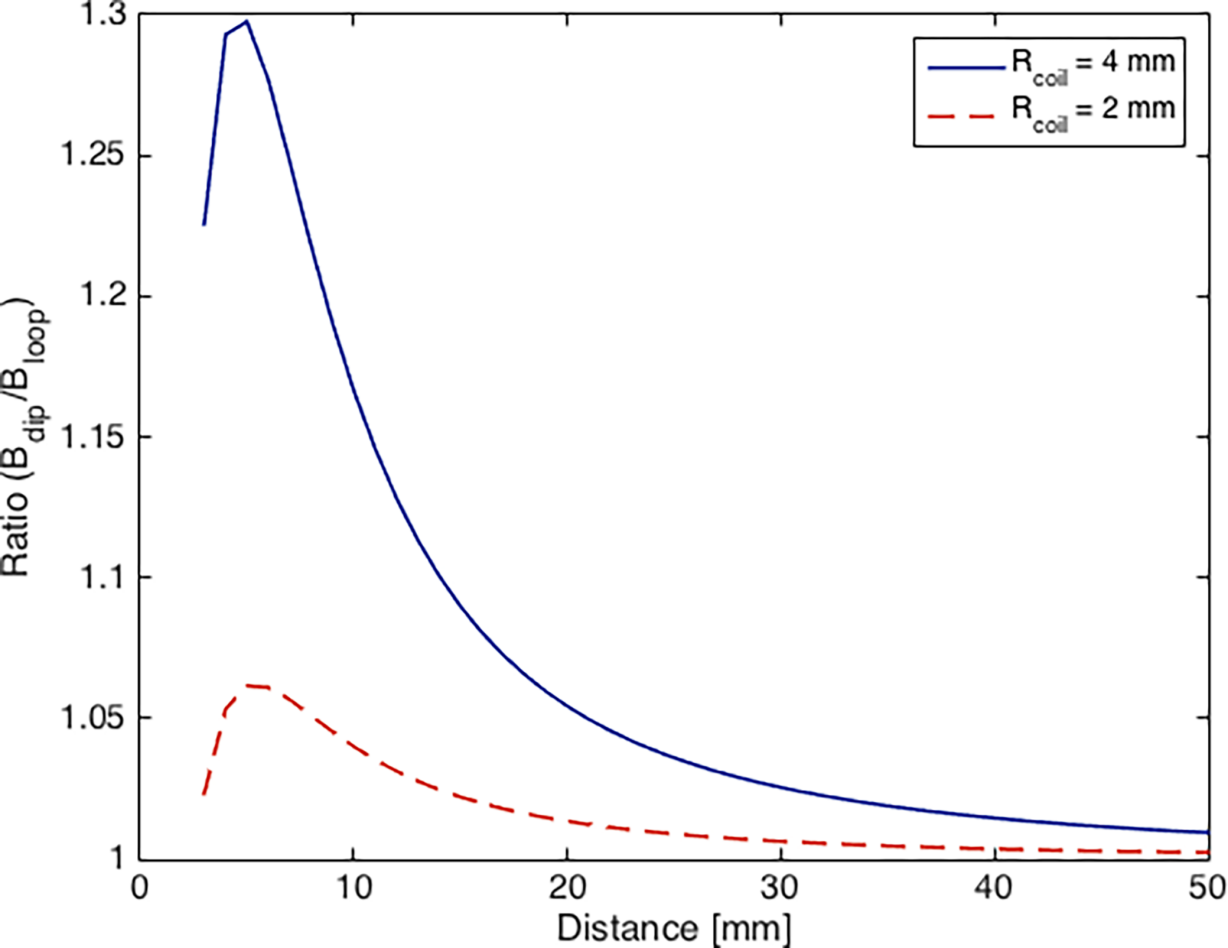
Accuracy of magnetic dipole approximation. Ratio between magnetic dipole approximation and exact solution of the magnetic field for coils with 2 mm (red, dotted) and 4 mm (blue) radii as a function of distance between sensor and coil.

Comparing the magnetic fields of a coil and the magnetic dipole approximation of the same (Fig 14) shows how the accuracy of the approximation depends on the distance between the coil and the sensor as well as on the size of the coil. The difference between actual and approximated magnetic fields is high as sensor and coil are close (up to 30% at 5 mm separation) and decreases significantly with distance. Placing the coils very close to the sensors could therefore lead to localization errors and should generally be avoided.

Since co-registration in commercial MEG systems is an over-determined problem, requirements on coil calibration and approximations of the fields they generate are not strict. As a result, the wire wound coils used typically (like the one in Fig 3) have high construction inaccuracies, making it hard to accurately model them. It would therefore be advisable to develop new coils that are optimized for this application. With printed circuit boards, for example, it should be possible to make smaller, more reliable coils. With more accurate coils it would be possible to use more sophisticated forward modeling of localization coils (e.g. elliptic integrals) to obtain better approximations of the magnetic fields.

## Conclusion

We introduced and evaluated a novel approach for localizing magnetometers for MEG using multiple localization coils. Our method is potentially beneficial for on-scalp MEG with flexible sensor arrays. We simulated the localization, showing that the approach works well in theory, and investigated the influence of different parameters on its accuracy. We found that, in order to provide high localization accuracy, position, orientation and calibration of the localization coils need to be determined accurately. SNR also has a strong impact on the localization accuracy and can be affected practically by magnetometer noise, the magnetic moment strengths of the localization coils (through the driving current) and the averaging time. Furthermore, the number of coils as well as their placement (i.e., their distance to the sensors) plays a role in the performance of the localization.

Of the parameters investigated the *a priori* knowledge about the localization coils is the main determining factor for the overall accuracy. With typical noise levels and even small localization coil arrays (4 coils in the local case and 10 in the full-head) the localization errors are well below 1 mm. Position errors on the order of the accuracy of a standard digitization device like the Polhemus Fastrak (1 mm) would, however, result in localization errors on the order of 2 mm.

We have proposed ways to tackle some of the challenges that we anticipate with the approach and improve its accuracy. These ideas should be further investigated and/or tested experimentally. Using the knowledge gained here we will be able to verify the technique in practical measurements and can try to optimize the localization of sensors for the accurate measurement and analysis of neuromagnetic activity in on-scalp MEG.

## References

[1] Hämäläinen MS, Hari R, Ilmoniemi RJ, Knuutila J, Lounasmaa OV. Magnetoencephalography–theory, instrumentation, and applications to noninvasive studies of the working human brain. Rev Mod Phys. 1993; 65(2):413–497.

[2] Supek S, Aine CJ, editors. Magnetoencephalography–From signals to dynamic cortical networks 1^st^ ed. Heidelberg: Springer 2014; ISBN: 978-3-642-33045-2.

[3] Schneiderman JF. Information content with low- vs. high-Tc SQUID Arrays in MEG Recordings: The Case for High-Tc SQUID- based MEG. J Neurosci Methods. 2014; 22:42–46. 10.1016/j.jneumeth.2013.10.007 24184856

[4] Boto E, Bowtell R, Krüger P, Fromhold TM, Morris PG, Meyer SS, et al On the potential of a new generation of magnetometers for MEG: A beamformer simulation study. PLOS ONE. 2016; doi: 10.1371/journal.pone.0157655 27564416 10.1371/journal.pone.0157655 PMC5001648

[5] Iivanainen J, Stenroos M, Parkkonen L. Measuring MEG closer to the brain: Performance of on-scalp sensor arrays. bioRxiv. 2016; doi: 10.1101/073585 10.1016/j.neuroimage.2016.12.048 PMC5432137 28007515

[6] Öisjöen F, Schneiderman JF, Figueras GA, Chukharkin ML, Kalabukhov A, Hedström A, et al High-Tc superconducting quantum interference device recordings of spontaneous brain activity: Towards high-Tc magnetoencephalography. Appl Phys Lett. 2012; 100:132601.

[7] Sander TH, Preusser J, Mhaskar R, Kitching J, Trahms L, and Knappe S. Magnetoencephalography with a chip-scale atomic magnetometer. Biomed Opt Express 2012; 3(5):981–990. doi: 10.1364/BOE.3.000981 22567591 10.1364/BOE.3.000981 PMC3342203

[8] Dammers J, Chocholacs H, Eich E, Boers F, Faley M, Dunin-Borkowski RE, et al Source localization of brain activity using helium-free interferometer. Appl Phys Lett. 2014; 104:213705.

[9] Xie M, Schneiderman JF, Chukharkin ML, Kalabukhov A, Whitmarsh S, Lundqvist D, et al High-Tc SQUID vs. low-Tc SQUID- based recordings on a head phantom: Benchmarking for magnetoencephalography. IEEE Trans Appl Supercond. 2015; 25:1601905.

[10] Boto E, Meyer SS, Shah V, Alem O, Knappe S, Kruger P, et al A new generation of magnetoencephalography: Room temperature measurements using optically-pumped magnetometers. NeuroImage. 2017; 149:404–414. doi: 10.1016/j.neuroimage.2017.01.034 28131890 10.1016/j.neuroimage.2017.01.034 PMC5562927

[11] Andersen LM, Oostenveld R, Pfeiffer C, Ruffieux S, Jousmäki V, Hämäläinen M, et al Similarities and differences between on-scalp and conventional in-helmet magnetoencephalography recordings. PLOS ONE 2017; doi: 10.1371/journal.pone.1712108 10.1371/journal.pone.0178602 PMC5524409 28742118

[12] Engels L, De Tiege X, Op de Beeck M, Warzée N. Factors influencing the spatial precision of electromagnetic tracking systems used for MEG/EEG source imaging. Clin Neurophysiol. 2010; 40(1):19–25. 10.1016/j.neucli.2010.01.002 20230932

[13] Oostenveld R, Fries P, Maris E, Schoffelen JM. FieldTrip: Open source software for advanced analysis of MEG, EEG, and invasive electrophysiological data. Comput Intell Neurosc. 2011; doi: 10.1155/2011/156869 21253357 10.1155/2011/156869 PMC3021840

[14] Uutela K, Taulu S, Hämäläinen M. Detecting and correcting for head movements in neuromagnetic measurements. NeuroImage 2001; 14:1424–1431. doi: 10.1006/nimg.2001.0915 11707098 10.1006/nimg.2001.0915

[15] Troebinger L, López JD, Lutti A, Bradbury D, Bestmann S, Barnes G. High precision anatomy for MEG. NeuroImage. 2014; 86:583–591. doi: 10.1016/j.neuroimage.2013.07.065 23911673 10.1016/j.neuroimage.2013.07.065 PMC3898940

[16] Meyer SS, Bonaiuto J, Lim M, Rossiter H, Waters S, Bradbury D, et al Flexible head-casts for high spatial precision MEG. J Neurosci Methods. 2017; 276: 38–45. doi: 10.1016/j.jneumeth.2016.11.009 27887969 10.1016/j.jneumeth.2016.11.009 PMC5260820

[17] Strom JH, Hömmen P, Drung D, Körber R. An ultra-sensitive and wideband magnetometer based on a superconducting quantum interference device. Appl. Phys. Lett. 2017; 110:072603.

[18] Faley MI, Poppe U, Urban K, Paulson DN, Fagaly RL. A new generation of the HTS multilayer DC-SQUID magnetometers and gradiometers. J Phys Conf Ser. 2006; 43:1199–1202.

[19] Kominis IK, Kornack TW, Allred JC, Romalis MV. A subfemtotesla multichannel atomic magnetometer. Nature. 2003; 422:596–599. doi: 10.1038/nature01484 12686995 10.1038/nature01484

[20] Colombo AP, Carter TR, Borna A, Jau YY, Johnson CN, Dagel AL, et al Four-channel optically pumped atomic magnetometer for magnetoencephalography. Optics Express. 2016; 24(14):15403–15416. doi: 10.1364/OE.24.015403 27410816 10.1364/OE.24.015403 PMC5025229

[21] Kakisaka Y, Wang ZI, Mosher JC, Dubarry AS, Alexopoulos AV, Enatsu R, et al Clinical evidence for the utility of movement compensation algorithm in magnetoencephalography: Successful localization during focal seizure. Epilepsy Res. 2012; 101(0):191–196. doi: 10.1016/j.eplepsyres.2012.03.014 22503605 10.1016/j.eplepsyres.2012.03.014 PMC3621737

[22] Urban E, Wakai RT. Optical sensor position indicator for neonatal MEG. IEEE Trans Biomed Eng. 2012; 59(1):255–262. doi: 10.1109/TBME.2011.2171960 22010142 10.1109/TBME.2011.2171960 PMC3244568

[23] Bardouille T, Krishnamurthy SV, Ghosh Hajra S, D’Arcy RCN. Improved localization accuracy in magnetic source imaging using a 3-D laser scanner. IEEE Trans Biomed Eng. 2012; 59(12):3491–3487. doi: 10.1109/TBME.2012.2220356 23033325 10.1109/TBME.2012.2220356

[24] Krishna Murthy SV, MacLellan M, Beyea S, Bardouille T. Faster and improved 3-D head digitization in MEG using Kinect. Front Neurosci. 2014; doi: 10.3389/fnins.2014.00326 25389382 10.3389/fnins.2014.00326 PMC4211394

[25] Knuutila J, Ahlfors S, Ahonen A, Hällström J, Kajola M, Lounasmaa OV, et al Large-area low-noise seven-channel dc SQUID magnetometer for brain research. Rev Sci Instrum. 1987; 58(11):2145–2156.

[26] Adjamian P, Barnes GR, Hillebrand A, Holliday IE, Singh KD, Furlong PL, et al Co-registration of magnetoencephalography with magnetic resonance imaging using bite-bar-based fiducials and surface-matching. Clin. Neurophysiol. 2004; 115:691–698. doi: 10.1016/j.clinph.2003.10.023 15036065 10.1016/j.clinph.2003.10.023

[27] Chella F, Zappasodi F, Marzetti L, Della Penna S, Pizzella V. Calibration of a multichannel MEG system based on the Signal Space Separation method. Phys. Med. Biol. 2012; 57:4855–4870. doi: 10.1088/0031-9155/57/15/4855 22797687 10.1088/0031-9155/57/15/4855

